# Effect of Antithrombin III Administration on the Prognosis of Severe Trauma Patients with Disseminated Intravascular Coagulation

**DOI:** 10.3390/healthcare11101476

**Published:** 2023-05-18

**Authors:** Jae Sik Chung, Myoung Jun Kim, Young Un Choi, Jun Gi Kim, Keum Seok Bae

**Affiliations:** Department of Surgery, Yonsei University Wonju College of Medicine, Wonju Severance Christian Hospital, Wonju 26426, Republic of Korea; gsjaesik@yonsei.ac.kr (J.S.C.); latilte21@gmail.com (M.J.K.); sangorilla@hanmail.net (Y.U.C.); melee_@yonsei.ac.kr (J.G.K.)

**Keywords:** antithrombin III, disseminated intravascular coagulation, prognosis, severe trauma

## Abstract

Background: We aimed to investigate the effects of antithrombin III administration on the prognosis of severe trauma patients with disseminated intravascular coagulation (DIC). Methods: Medical records of a total of 4023 patients who were admitted to the intensive care unit (ICU) at the single regional trauma center from January 2016 to December 2020 were retrospectively analyzed. After the exclusion of young patients (<15 years old), mild trauma (ISS < 16), non DIC, etc., a total of 140 patients were included in the study. These patients were classified into antithrombin III-administered and non-antithrombin III-administered groups. Clinical data, including laboratory findings, trauma- and ICU-related severity scores, prognosis (including length of hospital stay), and need for organ support, were retrospectively collected. We evaluated the characteristics of the two groups, and compared and analyzed the vital signs, laboratory findings, prognosis, and clinical outcomes of each group. With this, we analyzed the effect of antithrombin III administration in severe trauma patients with DIC. Results: Of the 140 patients, 61 were treated with antithrombin III. No significant difference was observed in the baseline characteristics between the two groups for initial laboratory results, initial vital signs, or trauma-related severity scores. The improvement of the sequential organ failure assessment (SOFA) score, a prognostic marker, was significantly greater in the administered group (*p* = 0.009). Additionally, the antithrombin-administered group showed a larger improvement in the SOFA score than the non-administered group (*p* = 0.002). However, there was no statistical difference between the two groups for the frequency or duration of organ support treatments (renal replacement therapy, mechanical ventilation), mortality, or length of hospital stay. Conclusion: Antithrombin III administration in severe trauma patients with DIC improved SOFA scores and aided in multi-organ dysfunction recovery. Appropriate indications should be studied to maximize the drug’s improvement effect in patients with severe trauma in the future.

## 1. Introduction

Trauma is the most common cause of death worldwide [1], and bleeding is the most common cause of death from trauma. Coagulopathies such as disseminated intravascular coagulation (DIC) and sequential multi-organ failure in patients with severe trauma are closely associated with poor outcomes [2]. The pathophysiology and clinical course of post-traumatic organ dysfunction are heavily influenced by whether a severe trauma patient has DIC and receives appropriate care [3].

There is no apparent strategy for appropriately managing DIC in patients with severe trauma, aside from providing overall supportive care and addressing the cause of disease and coagulopathy, despite numerous attempts. Research has revealed that sepsis-induced DIC (fibrinolytic type) and trauma-induced coagulopathy (TIC) share similar clinical phenotypes [2,4,5,6]. The diagnostic criteria for sepsis-induced DIC established by the International Society on Thrombosis and Hemostasis (ISTH) and the Japanese Association for Acute Medicine (JAAM) can be used to diagnose and predict the prognosis of trauma-related DIC [7]. Based on these previous studies, we hypothesized that the administration of antithrombin III might have a positive effect on trauma-related DIC, similar to sepsis-induced DIC.

In this study, we compared patients admitted to a single regional trauma center for severe trauma with DIC and an injury severity score (ISS) of ≥16, divided into an antithrombin III administration group and non-administration group. Additionally, we conducted a further investigation into the efficacy of antithrombin III in treating severely injured patients with DIC resulting from severe trauma. To evaluate the differences in clinical outcomes and changes in the sequential organ failure assessment (SOFA) score, which is commonly used to assess organ failure and predict patient prognosis in the intensive care unit (ICU), we analyzed data from both the antithrombin III administration group and the non-administration group. The purpose of this study was to examine the effects and potential benefits of antithrombin III in terms of treating severely injured patients with trauma-related DIC.

## 2. Methods

### 2.1. Patient Enrollment and Data Collection

In this study, we retrospectively analyzed data from patients admitted to the trauma ICU at a regional trauma center from January 2012 to December 2019. These patients included those who were directly admitted to the center following the trauma and those who were initially admitted to another medical facility and later transferred to our trauma center. Only the patients whose acute bleeding was deemed under control following a therapeutic intervention or an operation were included. Patients who underwent damage control surgery were excluded.

**Figure 1:**
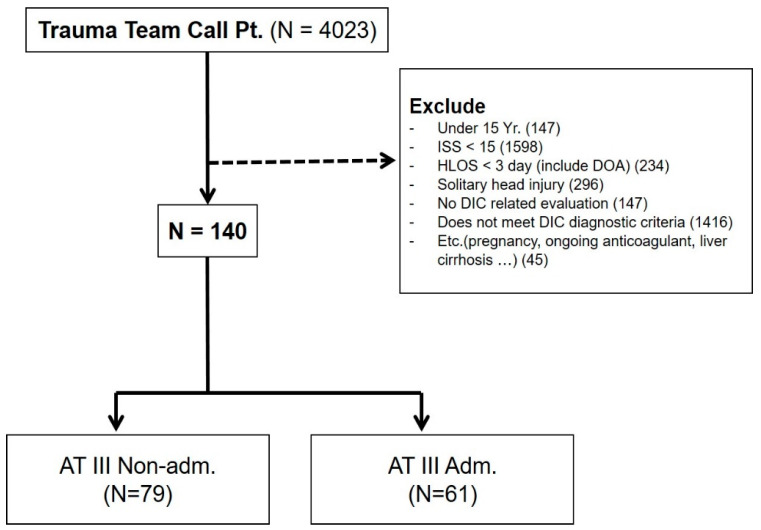
Patient enrollment in the study. One-hundred and forty patients were ultimately included. These patients were divided into the antithrombin III group (N = 61) and non-antithrombin III group (N = 79). Adm, administered; AT III, antithrombin III; DIC, disseminated intravascular coagulation; DOA, dead on arrival; HLOS, hospital length of stay; ISS, injury severity score; Pt, patient.

A total of 4023 trauma patients presenting to the trauma emergency department were initially identified, with 3883 trauma patients excluded using the following criteria: (1) aged < 15 years, (2) ISS ≤ 15, (3) died within 3 days after admission or before the administration of antithrombin III, (4) classified as having a solitary brain injury, (5) no laboratory test results to evaluate coagulopathy, (6) DIC was not confirmed based on the diagnostic criteria for DIC suggested by the Korean Society on Thrombosis and Hemostasis (KSTH) (Table 1), (7) pregnant women, (8) a history of chemo-radiation treatment owing to cancer, (9) a history of taking anticoagulants such as warfarin, aspirin, and clopidogrel, or (10) underlying medical conditions that could cause coagulopathy such as liver cirrhosis. After applying the exclusion criteria, 140 patients were selected for inclusion in this study (Figure 1). These patients were divided into the antithrombin III group (N = 61) and non-antithrombin III group (N = 79). 

The product administered in this study was antithrombin III Human 500 IU by GC Pharma, a domestic pharmaceutical company. The product is administered at an initial dose of 1000–2000 units and then at a maintenance dose of 2000–3000 units per day (500 units every 4–6 h). The administration method is as follows. After dissolving the antithrombin dry concentrate in 10 mL of saline for injection per 500 units, it is slowly intravenously injected. The medical records of the enrolled patients were reviewed to check their history of product usage.

### 2.2. Clinical Variables

Each patient’s basic characteristics such as sex, age, height, weight, and medical history, abbreviated injury scale (AIS) score for each body system (head and neck, face, chest, abdomen, extremity, and external), ISS, revised trauma score (RTS), surgery or intervention records, initial vital signs (systolic blood pressure, diastolic blood pressure, pulse rate, and respiration rate), worst vital signs within 2 h after the hospital admission, and Glasgow coma scale (GCS) score were reviewed.

Various laboratory test results were reviewed. These included cell blood counts (CBCs), including white blood cell (WBC), hemoglobin (Hb), and platelet (Plt) count; routine chemistry test results, including blood urea nitrogen (BUN), creatinine, and total bilirubin levels; blood coagulation test results, including prothrombin time (PT) and activated partial thromboplastin time (aPTT); arterial blood gas analysis (ABGA) results, including pH, pO_2_, pCO_2_, O_2_ saturation, BE (base excess), and serum lactate; and DIC-related test results, including fibrinogen degradation product (FDP), D-dimer, and antithrombin III levels.

The amount of blood transfusion, vasopressor concentration and duration, total duration of hospital stay, duration of ICU stay, death, incidence and duration of mechanical ventilation, and incidence and duration of renal replacement therapy (including continuous renal replacement therapy and conventional hemodialysis) were investigated and compared between the two groups.

The SOFA score of each patient was reviewed. SOFA scores from the day of hospital admission to 15 days after the admission were reviewed. Changes in SOFA scores from the day of DIC diagnosis to 15 days after the admission or discharge/death (delta SOFA) were also reviewed.

These variables were compared between patients with severe trauma accompanied by DIC who were treated with antithrombin III and those who were not.

### 2.3. Statistical Analysis

Statistical analysis of the investigated items was performed using SPSS Statistics 25.0 (IBM Corp., Armonk, NY, USA). Categorical data are presented as numbers (%); these data were compared using the chi-square test or Fisher’s exact test. Continuous variables are expressed as mean ± standard deviation or as median and interquartile range; these data were compared between groups using Student’s *t*-test or the Mann–Whitney U test. Using these tests, variables showing a significant difference between the groups were identified. A *p*-value of less than 0.05 was considered statistically significant. 

## 3. Results

A total of 140 patients with severe trauma based on the inclusion and exclusion criteria who satisfied the DIC criteria were enrolled in this study. Of these, 61 patients were administered antithrombin III, and 79 patients were not administered antithrombin III. The 28-day and overall mortalities among the enrolled patients were 13.6% and 27.86%, respectively. The mean total dose of antithrombin III administered was 7562.5 ± 2708.7 units, and the mean length of the administration period was 5.48 ± 1.14 days. 

Table 2 shows the baseline characteristics of the two groups. No significant differences were found in age, sex, body mass index, or underlying medical diseases between the two groups. No significant differences in the measures of injury severity such as the AIS score of each system, ISS, or RTS were found between the two groups. No significant differences were found in the incidence of surgery between the groups. The incidence of radiologic therapeutic intervention was significantly higher in the antithrombin III group than in the non-antithrombin III group.

Table 3 summarizes the worst vital signs within 2 h after admission following trauma and initial laboratory findings. No significant differences in vital signs or GCS were found between the two groups. No significant differences were found in most of the initial results regarding CBC, routine chemistry, ABGA, and DIC-related laboratory markers between the groups.

### 3.1. Comparison of Change in SOFA Score between the Two Groups

**Figure 2:**
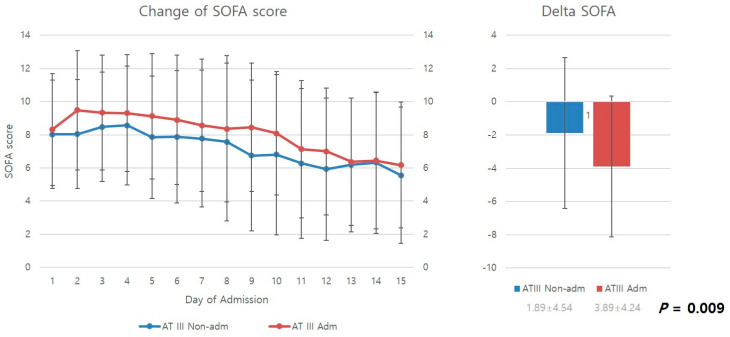
Change in SOFA score and delta SOFA. AT III, antithrombin III; Adm, administration; Non-admn, non-administration; SOFA, sequential organ failure assessment.

Figure 2 compares the mean SOFA scores from the day of admission to the 15th in-hospital day of stay. SOFA scores decreased over time for both the antithrombin III and non-antithrombin III groups as patients received ICU care. However, upon examination of delta SOFA scores, representing changes in SOFA scores from the day of DIC diagnosis to the 15th in-hospital day of stay, it was found that the antithrombin III group had significantly higher delta SOFA scores than the non-antithrombin III group, showing a greater improvement in SOFA scores (*p* = 0.009).

**Figure 3:**
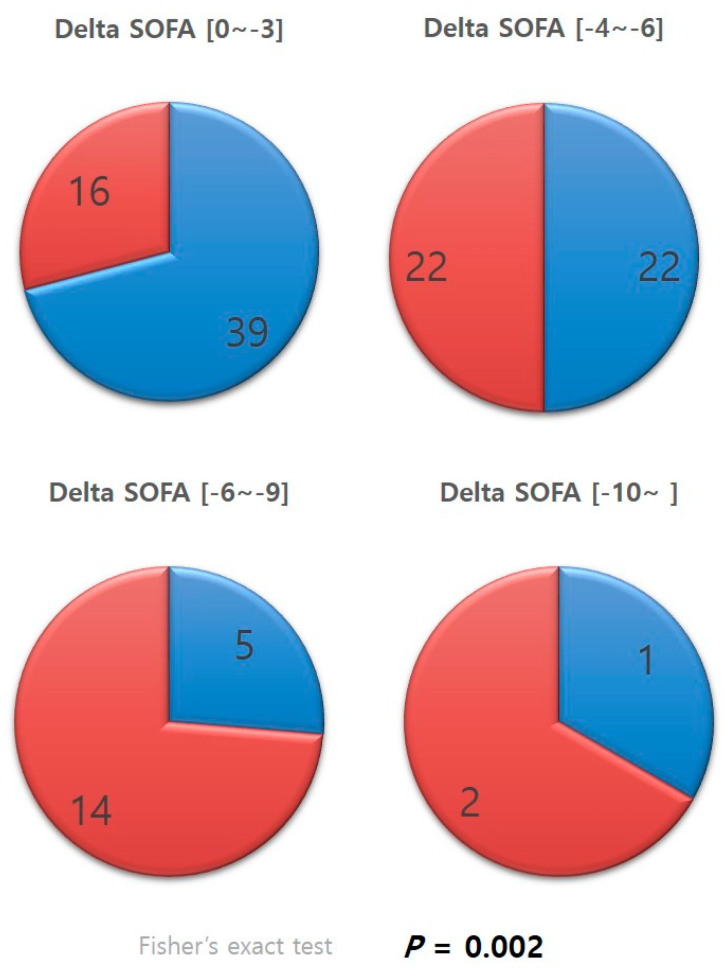
Categorized delta SOFA. SOFA, sequential organ failure assessment.

Patients whose delta SOFA scores were negative, in other words, patients who showed improvements in SOFA scores, were categorized according to the level of improvement (Figure 3). The distribution of the categories in each group was examined. A significantly greater percentage of patients in the antithrombin III group were in the categories with greater improvements in SOFA scores than that of those in the non-antithrombin III group (*p* = 0.002).

### 3.2. Comparison of Transfusion Requirements between the Two Groups

The antithrombin III group showed significantly greater red blood cell (RBC) usage than the non-antithrombin III group during the first week. The two groups showed no significant difference in RBC usage thereafter. No significant differences in fresh frozen plasma (FFP) and platelet concentrate (Plt) were found between the two groups (Table 4).

### 3.3. Comparison of Organ Support Requirements between the Two Groups

The antithrombin III group had a shorter mean duration of mechanical ventilation than the non-antithrombin III group (16.3 days vs. 17.5 days, respectively); however, it was not significantly different. The two groups showed no significant differences in the incidence or duration of renal replacement therapy (including continuous renal replacement therapy and hemodialysis) either. Additionally, they showed no significant difference in the duration of use of intravenous vasopressors, such as norepinephrine or vasopressin (Table 5).

### 3.4. Comparison of Clinical Outcomes between the Two Groups

The antithrombin III group showed a shorter mean duration of ICU stay than the non-antithrombin group, but the difference was not statistically significant. The two groups showed no significant difference in the total duration of hospital stay. The antithrombin III group showed a lower 28-day mortality than the non-antithrombin III group (13.1% vs. 14.7%), but this difference was not statistically significant either. Furthermore, no significant difference in overall mortality between the two groups was found (Table 6). 

## 4. Discussion

Immediate post-trauma care, including bleeding control, is the most important determinant of prognosis for patients with severe trauma [8], followed by prevention and treatment of organ dysfunction, including coagulopathy [9]. Therefore, trauma research on the prevention and appropriate management of organ failure such as coagulopathy, including DIC, is critical during treatment in the ICU.

However, planned research on such topics is difficult given the characteristics of trauma patients. The mechanisms of trauma vary between patients, and a single traumatic event can damage multiple organs regardless of its mechanism [10]. Furthermore, multiple organ damage can easily progress to multi-organ failure. Owing to the heterogenic characteristics of trauma patients, research on the effect of antithrombin III on coagulopathy and DIC features associated with trauma is more difficult than research on sepsis-induced DIC, for which numerous studies have already been conducted.

Despite this difficulty, this study is valuable because it analyzed a sufficiently large number of cases (N = 140) that satisfied specific conditions, such as having trauma with an ISS ≥ 16, satisfying the DIC diagnostic criteria, and receiving ICU care.

This study demonstrated that appropriate ICU management can alleviate and prevent DIC-associated organ dysfunction in patients with severe trauma exhibiting DIC features based on SOFA scores, which are a measure of organ dysfunction [11]. The improvements identified in the mean SOFA scores support this finding (Figure 2).

This study examined improvements in SOFA scores (delta SOFA scores). Delta SOFA is a measure of changes in SOFA scores. Iba et al. reported that delta SOFA scores can be used to examine the treatment effect and prognosis of critically ill patients [12]. Delta SOFA scores are also strongly associated with outcome variables such as mortality in patients with DIC [13]. In this study, the antithrombin III group showed a significantly higher delta SOFA score than the non-antithrombin III group (−3.89 ± 4.24 vs. 1.89 ± 4.54) (*p* = 0.009). This result demonstrates that antithrombin III administration effectively prevents organ failure and promotes recovery from organ dysfunction in patients with progressive organ dysfunction resulting from DIC features caused by severe trauma.

The difference between the two groups was increasingly evident after categorizing patients according to their delta SOFA scores (Figure 3). Cases with negative delta SOFA values, in other words, cases with improvements in SOFA scores, were divided into subgroups according to the level of improvement. A significantly higher percentage of patients in the antithrombin III group were found to belong to subgroups with larger delta SOFA values than those in the non-antithrombin III group (*p* = 0.002). This result suggests that antithrombin III has a positive or booster effect, powerfully and effectively promoting recovery from multi-organ dysfunction resulting from DIC. Several studies support our findings, reporting that antithrombin III administration had a beneficial effect on various outcome variables, and the effect was even more evident for sepsis-induced DIC [14,15,16]. Antithrombin III supplements may promote recovery from organ dysfunction by acting as a regulator of adverse chain reactions in the vicious cycle of uncontrolled thrombosis activation and consumption of coagulation factors caused by DIC, leading to overwhelmed thrombosis and the formation of microthrombi, culminating in organ dysfunction [17,18]. Antithrombin III has been shown to reduce complications in trauma animal models [19] and reduce the duration of organ failure in trauma patients [20].

Unlike existing studies on the use of antithrombin III for sepsis-induced DIC, the present study did not observe significant differences in various clinical outcomes between the patients administered antithrombin III and those who were not. Although microscopic improvements in organ dysfunction were observed, the improvements were not as evident at the macroscopic level. Antithrombin III administration did not lead to significant improvements in the amount of blood transfusion, duration of ICU stay, total duration of hospital stay, or incidence and duration of renal replacement or mechanical ventilation [20]. More research is needed to establish precise indications for antithrombin III administration in patients with severe trauma exhibiting DIC features until it can be used to improve clinical outcomes or until the effect of antithrombin III can be maximized, as shown in Figure 3. Although much about the physiological mechanism of trauma-induced coagulopathy has been revealed through research, clinical diagnostic criteria for TIC are yet to be established. However, as the clinical similarity between TIC and sepsis-induced DIC (fibrinolytic type) has been reported, management guidelines are starting to be established for TIC [21,22]. Although the use of antithrombin III for septic DIC has considerably decreased since the KyberSept trial [23], this trial became a driving force for re-examining and researching the diagnostic criteria for DIC, as well as the dose and duration of antithrombin III use. Antithrombin III indications and methods of administration in trauma care should be clarified through further prospective research.

In this study, the KSTH criteria were used to diagnose DIC. The KSTH criteria have been shown to be more useful for predicting prognoses than the ISTH or JAAM DIC diagnostic criteria [24]. Additionally, many clinicians follow the KSTH criteria when administering antithrombin III as the medical fees for antithrombin III treatment are determined according to the criteria.

The present study has some limitations, among which is its retrospective design. First, the time and interval of antithrombin III administration was not perfectly controlled. However, most patients were administered antithrombin III immediately after receiving a diagnosis of DIC based on their medical records, and antithrombin III was administered at the dose and interval specified in a guideline. Patients who were not administered antithrombin III according to the guideline were excluded from the study. Second, some data were missing from the data set. Nonetheless, the two groups in this study did not show significant differences in most baseline characteristics; thus, the missing data did not have a significant impact on the interpretation of the results. Third, while this study had a relatively larger sample size than other studies with a similar design, it used data from a single institution, and the results derived from such data may not directly apply to other institutions. Fourth, as trauma patients tend to have multiple medical conditions with various severities, the coagulation and fibrinolytic status of trauma patients drastically change during their clinical course [25]. Fifth, the use of the KSTH criteria for the diagnosis of DIC can be applied as a limitation to generalizing the study’s results to other ethnic groups or countries. Therefore, it is thought that a prospective study based on more commonly used diagnostic criteria is needed to strengthen the validity of this research.

## 5. Conclusions

In conclusion, antithrombin III administration provided during ICU care for patients with severe trauma exhibiting DIC features significantly improves SOFA scores. A significant difference in SOFA scores was found between the group that was administered antithrombin III and the group that was not. Antithrombin III administration can improve SOFA scores more powerfully and effectively. This result demonstrates that antithrombin III can help prevent and reduce organ dysfunction caused by DIC in patients with severe trauma.

In this study, antithrombin III administration did not improve clinical outcomes such as the length of stay, mortality, organ support treatment requirement, or transfusion requirement. Through further prospective research, clear indications of antithrombin III administration should be established to maximize the effect of this drug, and this should be discussed in follow-up studies.

## Figures and Tables

**Table 1:** Diagnostic criteria for DIC of the KSTH.

	Score	KSTH
Platelets, ×10^3^/L	0	>100
	1	≤100
PT, sec	0	<3
	1	≥3
aPTT, sec	0	<5
	1	≥5
Fibrin-related marker, μg/mL	0	No increase
	1	Increase
Fibrinogen, g/L	0	>1.5
	1	≤1.5
Total		DIC ≥ 3

**Table 2:** Baseline characteristics of patients.

	AT III Non-Adm. (N = 79)	AT III Adm. (N = 61)	*p*-Value
Age (years), mean ± SD	56.41 ± 16.29	58.21 ± 16.48	0.518
Gender, N (%)			0.168
Male	51 (64.6)	46 (75.4)	
Female	28 (35.4)	15 (24.6)	
BMI, mean ± SD	24.15 ± 4.86	23.36 ± 3.07	0.267
Underlying Dz (%)			
HTN	19 (24.1)	14 (23)	0.879
DM	11 (13.9)	8 (13.1)	0.89
CRF	1(1.3)	0 (0)	0.378
CAOD	0 (0)	1 (1.6)	0.253
Cancer	8 (10.1)	2 (3.3)	0.119
AIS, mean ± SD			
Head and neck	1.72 ± 1.87	2.08 ± 2.01	0.275
Face	0.44 ± 0.78	0.51 ± 0.91	0.649
Chest	1.87 ± 1.62	2.18 ± 1.71	0.28
Abdomen	2.04 ± 1.58	1.56 ± 1.56	0.075
Extremities	2.08 ± 1.74	1.98 ± 1.69	0.753
External	0.06 ± 0.56	0.03 ± 0.18	0.684
ISS, mean ± SD	27.38 ± 12.04	28.15 ± 9.33	0.681
RTS, mean ± SD	5.715 ± 1.796	6.203 ± 1.806	0.115
Intervention (%)	8 (10.1)	14 (23)	0.039
Operation (%)	70 (88.6)	53 (86.9)	0.757

AT III = antithrombin III, Adm. = administration, SD = standard deviation, BMI = body mass index, Dz = disease, HTN = hypertension, DM = diabetes mellitus, CRF = chronic renal failure, CAOD = coronary artery occlusive disease, AIS = abbreviated injury scale, ISS = injury severity score, RTS = revised trauma score.

**Table 3:** Comparison of clinical parameters.

	AT III Non-Adm. (N = 79)	AT III Adm. (N = 61)	*p*-Value
V/S_worst (within 2 h), mean ± SD			
SBP_worst (mmHg)	70.9 ± 17.4	75.1 ± 23.9	0.235
DBP_worst (mmHg)	38.9 ± 10.9	39.6 ± 12.5	0.74
PR_worst	154.0 ± 148.9	150.26 ± 133.1	0.877
RR_worst	29.3 ± 11.6	38.3 ± 28.8	0.012
GCS_worst, mean ± SD	5.7 ± 4.2	5.9 ± 4.1	0.729
Lab_initial, mean ± SD			
WBC (10^9^/L)	14.9 ± 6.8	13.8 ± 7.3	0.341
Hb (g/dL)	10.4 ± 2.2	10.8 ± 2.5	0.301
Plt (10^9^/L)	171.7 ± 66.9	175.3 ± 87.5	0.785
Cr (mg/dL)	1.1 ± 0.8	1.0 ± 0.4	0.158
T.bil (g/dL)	0.7 ± 0.7	0.7 ± 0.6	0.981
aPTT (sec)	37.3 ± 13.9	47.3 ± 67.1	0.194
PT(INR)	1.4 ± 0.3	1.4 ± 0.5	0.999
pH	7.283 ± 0.147	7.316 ± 0.138	0.188
pO_2_	151.6 ± 58.9	154.7 ± 65.3	0.773
pCO_2_	33.3 ± 10.3	31.4 ± 7.1	0.212
O_2_ sat. (%)	95.3 ± 10.7	95.5 ± 12.8	0.895
BE (mmol/L)	−10.2 ± 6.0	−9.0 ± 6.4	0.262
Lactate (mmol/L)	5.6 ± 3.5	5.6 ± 3.6	0.99
FDP (μg/mL)	229.3 ± 184.9	170.2 ± 137.8	0.039
Fibrinogen (mg/dL)	151.6 ± 103.7	168.9 ± 98.3	0.32
D-Dimer (ng/mL)	19,667.1 ± 14,491.7	19,708.9 ± 17,012.9	0.987
AT III (%)	63.6 ± 17.7	58.5 ± 20.5	0.119

AT III = antithrombin III, Adm. = administration, V/S = vital sign, h = hour, SD = standard deviation, SBP = systolic blood pressure, DBP = diastolic blood pressure, PR = pulse rate, RR = respiration rate, GCS = Glasgow coma scale, Lab. = laboratory, WBC = white blood cell, Hb = hemoglobin, Plt = platelet count, Cr = creatinine, T.bil = total bilirubin, aPTT = activated partial thromboplastin time, PT = prothrombin time, INR = international normalized ratio, pO_2_ = partial pressure of oxygen, pCO_2_ = partial pressure of carbon dioxide), O_2_ sat. = oxygen saturation of arterial blood, BE = base excess, FDP = fibrinogen degradation product.

**Table 4:** Change in transfusion requirement.

	AT III Non-Adm. (N = 79)	AT III Adm. (N = 61)	*p*-Value
RBC day 1–3, mean ± SD	1.9 ± 2.0	3.0 ± 2.6	0.004
RBC day 3–7, mean ± SD	1.1 ± 2.0	1.9 ± 2.2	0.025
RBC day 7–14, mean ± SD	0.9 ± 2.8	1.18 ± 2.5	0.469
RBC day 14–28, mean ± SD	0.6 ± 2.2	1.2 ± 3.7	0.239
FFP day 1–3, mean ± SD	1.3 ± 1.5	1.9 ± 2.1	0.053
FFP day 3–7, mean ± SD	0.6 ± 1.3	1.0 ± 1.5	0.058
FFP day 7–14, mean ± SD	0.3 ± 1.8	0.6 ± 1.7	0.480
FFP day 14–28, mean ± SD	0.3 ± 1.8	0.5 ± 2.7	0.519
Plt day 1–3, mean ± SD	0.9 ± 1.1	1.2 ± 1.2	0.133
Plt day 3–7, mean ± SD	0.6 ± 0.9	1 ± 1.9	0.071
Plt day 7–14, mean ± SD	0.1 ± 0.3	0.4 ± 1.3	0.025
Plt day 14–28, mean ± SD	0.1 ± 0.4	0.2 ± 0.7	0.339

AT III = antithrombin III, Adm. = administration, RBC = red blood cell, SD = standard deviation, FFP = fresh frozen plasma, Plt = platelet concentrate.

**Table 5:** Need for organ support treatment of two groups.

	AT III Non-Adm. (N = 79)	AT III Adm. (N = 61)	*p*-Value
Incidence of mechanical ventilation (%)	66 (88)	56 (93.3)	0.297
Duration of mechanical ventilation (day), mean ± SD	17.5 ± 18.9	16.3 ± 12.3	0.694
Incidence of renal replacement therapy (%)	14 (18.7)	15 (24.6)	0.402
Duration of renal replacement therapy (day), mean ± SD	20.5 ± 24.7	23.8 ± 16.4	0.673
Duration of using iv vasopressor (day), mean ± SD	5.0 ± 12.8	5.2 ± 5.8	0.912

AT III = antithrombin III, Adm. = administration, SD = standard deviation, iv = intra-venous.

**Table 6:** Comparison of length of stay and mortality.

	AT III Non-Adm. (N = 79)	AT III Adm. (N = 61)	*p*-Value
ICU LOS (day), mean ± SD	23.0 ± 33.6	21.4 ± 17.9	0.749
H-LOS (day), mean ± SD	61.8 ± 52.1	73.1 ± 71.4	0.29
28-day mortality	11 (14.7)	8 (13.1)	0.795
Overall mortality	14 (18.7)	15 (24.6)	0.402

AT III = antithrombin III, Adm. = administration, SD = standard deviation, ICU = intensive care unit, LOS = length of stay, H-LOS = hospital length of stay (overall length of stay).

## Data Availability

The datasets used and/or analyzed during the current study are available from the corresponding author upon reasonable request.

## Abbreviations

ABGA, arterial blood gas analysis; AIS, abbreviated injury scale; aPTT, activated partial thromboplastin time; BE, base excess; BUN, blood urea nitrogen; CBCs, cell blood counts; DIC, disseminated intravascular coagulation; ED, emergency department; FDP, fibrinogen degradation product; FFP, fresh frozen plasma; Hb, hemoglobin; ICU, intensive care unit; ISS, injury severity score; ISTH, International Society on Thrombosis and Hemostasis; JAAM, Japanese Association for Acute Medicine; KSTH, Korean Society on Thrombosis and Hemostasis; Plt, platelet; PT, prothrombin time; RBC, red blood cell; RTS, revised trauma score; SOFA, sequential organ failure assessment; TIC, trauma-induced coagulopathy; WBC count, white blood cell count.
